# Structural Disorder in Eukaryotes

**DOI:** 10.1371/journal.pone.0034687

**Published:** 2012-04-05

**Authors:** Rita Pancsa, Peter Tompa

**Affiliations:** 1 VIB Department of Structural Biology, Vrije Universiteit Brussel, Brussels, Belgium; 2 Institute of Enzymology, Hungarian Academy of Sciences, Budapest, Hungary; Hungarian Academy of Sciences, Hungary

## Abstract

Based on early bioinformatic studies on a handful of species, the frequency of structural disorder of proteins is generally thought to be much higher in eukaryotes than in prokaryotes. To refine this view, we present here a comparative prediction study and analysis of 194 fully described eukaryotic proteomes and 87 reference prokaryotes for structural disorder. We found that structural disorder does distinguish eukaryotes from prokaryotes, but its frequency spans a very wide range in the two superkingdoms that largely overlap. The number of disordered binding regions and different Pfam domain types also contribute to distinguish eukaryotes from prokaryotes. Unexpectedly, the highest levels – and highest variability – of predicted disorder is found in protists, i.e. single-celled eukaryotes, often surpassing more complex eukaryote organisms, plants and animals. This trend contrasts with that of the number of domain types, which increases rather monotonously toward more complex organisms. The level of structural disorder appears to be strongly correlated with lifestyle, because some obligate intracellular parasites and endosymbionts have the lowest levels, whereas host-changing parasites have the highest level of predicted disorder. We conclude that protists have been the evolutionary hot-bed of experimentation with structural disorder, in a period when structural disorder was actively invented and the major functional classes of disordered proteins established.

## Introduction

Deciphering protein structures has been instrumental in understanding the molecular principles of life. Yet, the recent most exciting development in structural biology is the recognition that many proteins (intrinsically disordered proteins, IDPs) or regions of proteins (intrinsically disordered regions, IDRs) exist and function without a well-defined structure [1], [2], [3]. The existence and functioning of IDPs/IDRs demand a radical extension of the structure-function paradigm to encompass their non-conventional functional modes. The functional advantages of structural disorder are manifested either directly, in functions termed entropic chains, or in molecular recognition, in the form of adaptable binding [4], uncoupling specificity from binding strength [5] or increasing the speed of interactions [6], [7], among others. Due to these advantages, an elevated level of structural disorder can be found in proteins involved in signaling and regulation, and structural disorder is often associated with disease, such as cancer and neurodegeneration [7].

The functional advantages and functional types of IDPs/IDRs predisposes them for roles in complex organisms, in broad agreement with the observed phylogenetic distribution of structural disorder [8], [9], [10]. Based on previous studies on a few genomes available at the time (usually comparing predicted disorder in 4–5 eukaryotes to bacteria and archea), it has become generally accepted that structural disorder is significantly higher in eukarytoes than in prokaryotes, expressed by the notion that structural disorder correlates with complexity. Besides these comparative studies, the level of disorder was only addressed in particular phylogenetic groups, such as bacteria [11], [12], archaea [13] or a few protists within eukaryotes [14], [15]. More recent studies presented large-scale analyses, without trying to derive general conclusions [16]. The suggested correlation with complexity was directly addressed for organisms of known complexity measures (number of different cell types) [17]. It was found that disorder has a tendency to increase in evolution, but its correlation with complexity within eukaryotes is marginal.

Therefore, even these limited studies have raised certain caveats to the above generalizations, and suggested exceptions to the seemingly simple and general rule. For example, studies on the distribution of predicted structural disorder in prokaryotes has shown wide variations as a function of growth temperature, with mesophiles an thermophyles covering a very broad range from ∼1.5% to ∼25% but hyperthermophiles having much less [11], [12]. Archaea were also found to show wide disorder distribution, with strong genomic variations depending on habitat and lifestyle [13]. Turning to eukaryotes, Apicomplexan protists - single-celled eukaryotes - have shown unexpectedly high levels of predicted disorder, way exceeding that of apparently more complex metazoan organisms [14]. Similar conclusions were drawn in a study of a handful of early-branching protists [15], which again showed a high level of predicted disorder surpassing the average of eukaryotic proteins in SwissProt. It was raised that structural disorder may be associated with the parasitic lifestyle of these organisms.

As apparent from this short overview, structural disorder has not been systematically and comparatively analyzed in eukaryotes. Apparently, one of the reasons is a very fast advance in sequencing efforts, due to which about two-thirds of known eukaryotic genomes became available in the past five years or so. Furthermore, the results of distinct studies are hard to compare, because they rely on different disorder predictors usually based on different principles and having significantly different rates of confidence [18], [19], [20]. In addition, often related but different measures of structural disorder (frequency of disordered residues, frequency of proteins with a long IDR, or frequency of mostly disordered proteins) are applied, which again impedes comparisons and sound generalizations. Therefore, we decided to predict and compare structural disorder in 194 available eukaryotic proteomes (and 87 reference prokaryotes) with the IUPred algorithm [21], [22]. We extended and complemented these calculations with predictions of the prevalence of Pfam domains and comparing disorder within and outside domains, because: i) disordered regions often harbor binding motifs for domains [23], ii) disordered regions often function by acting as linkers between flanking domains, and iii) structural disorder may also be present in Pfam domains themselves [24]. The novel data on the phylogenetic distribution of structural disorder, Pfam domain types, and their varied correlation in different types of species refine previous limited generalizations and provide novel insight into the evolutionary and functional implications of structural disorder.

## Methods

### Eukaryotic, prokaryotic and archaeal proteomes

Most of the eukaryotic proteomes were downloaded from the complete proteome set of the UniProt database [25], and some additional ones from the RefSeq database [26]. To avoid redundancy, we usually used only one proteome for species for which multiple strains are available (such as, in the case of *S. cerevisiae*, for example). In the case of non-pathogenic higher-order organisms, we only used one representative proteome for several very closely related species within one genus (such as in case of the Drosophila genus); for pathogens, we kept the proteomes of all species. For all the species analyzed, we indicate the source and date of actual downloading in Supplementary Table S1.

For a comparison, proteomes of all the reference Bacteria (69) and Archaea (18) were also downloaded from the UniProt database (cf. Supplementary Table S2 and S3, respectively). In case of the eukaryotic proteomes, the downloaded sequence data also contained all the known isoforms. To avoid redundancy, all the proteomes were filtered for 90% sequence identity with the CD-HIT V4.5.4 program [27]. For every filtered proteome, the number of proteins and the average protein length was calculated. Proteomes thus filtered were used for further analysis.

### Prediction of structural disorder and disordered binding sites

Structural disorder and disordered binding sites were predicted by the ANCHOR algorithm [16] which incorporates the IUPred algorithm for disorder prediction [21], [22]. From predicted IUPred disorder scores, we calculated distinct measures of disorder. First, the average disorder score for all proteins was calculated by averaging the value of disorder scores for individual residues across the entire protein. The ratio of disordered residues was calculated as the percentage of residues within the protein with a disorder score ≥0.5. This value was also calculated for regions identified as Pfam domains (for prediction of Pfam domains, see next section) and also for regions outside Pfam domains. Here we divided all the proteins of the proteomes in which at least one Pfam family, domain or repeat was predicted into two parts, one containing all the predicted Pfam domains, the other containing the rest of the protein. For these we calculated the ratio of disordered residues separately. The values calculated for the regions outside the domains were grouped together with the values for those proteins in which no Pfam domains were found. From all the values determined, measures for entire proteomes were calculated as follows. The average disorder of proteins was averaged for the whole proteome, yielding the value referred to as average disorder in the proteome. The ratio of disordered residues (disorder score ≥0.5) was also averaged for all proteins, termed thereafter as the average ratio of disordered residues in proteins. The ratio of proteins with at least one long (≥30 consecutive residues with a score ≥0.5) disordered region and the ratio of amino acids within these regions was also calculated. From the results of binding-site predictions by ANCHOR, only those sites were kept, which are marked as “real” by ANCHOR. The overall number of disordered binding sites and the average number of disordered binding sites per protein was calculated for every proteome. All calculated data are found in Supplementary Table S1, S2, and S3 for Eukaryotes, Bacteria and Archaea, respectively.

### Prediction of Pfam domains

With the PfamScan method [28], all Pfam-A families, domains and repeats were predicted for every protein in our datasets. Pfam-B domains were neglected because of much lower quality of the underlying HMM profiles. The total number of different types of domains/families/repeats was calculated for every proteome. In the text we refer to these regions as Pfam domains, irrespective of their actual type (i.e. family, domain or repeat). All the calculated data are found in Supplementary Table S1, S2, and S3 for Eukaryotes, Bacteria and Archaea, respectively. The ratios of disordered residues as defined in the previous section were also calculated and averaged for the Pfam domains and also regions outside domains.

### Phylogenetic groups

There are various existing phylogenies for eukaryotes, we implemented our data with the one used by UniProt except for the Opisthoconta kingdom, which contains Fungi and Metazoa as well as some other protists. Because of the large amount of species in these two groups, we decided to handle them separately from the remaining two species in Opisthoconta, so in the figures only the two species are marked to be Opisthoconta and the others in this kingdom are separated to Fungi and Metazoa groups.

## Results

### Structural disorder, domains and motifs in the three superkingdoms of life

**Figure 1:**
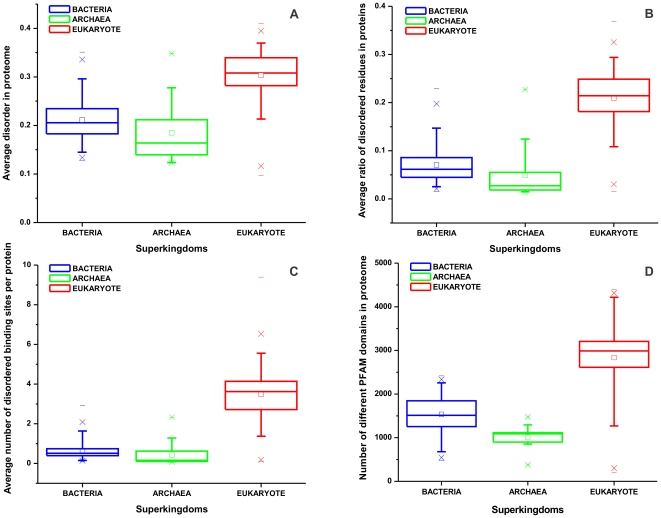
Structural features of the proteomes in the three superkingdoms. Structural disorder was predicted by the IUPred algorithm [21], [52] for all the proteins in the proteomes collected; the average disorder (**A**) and the ratio of disordered residues (**B**) were calculated for the three superkingdoms, Bacteria, Archaea and Eukaryote. Disordered binding sites (**C**) were predicted by the ANCHOR method [16] and averaged for all proteins in all proteomes in the three superkingdoms. The search for Pfam domains (**D**) was carried out by the PfamScan algorithm [28]. The number of different Pfam domains was calculated in all proteomes and averaged in the three superkingdoms. On every panel, the horizontal line in the box shows the median of the data, the mean is indicated by a small square, and the upper and lower edge of the box indicates the 75 and 25% of the data, respectively. The upper and lower error bars show the 90 and 10% of the data respectively, the upper and lower cross represents 99% and 1% of the data, while the maximum and minimum value within the dataset is indicated by short horizontal lines.

Based on much more species than in previous analyses, we first asked if the distribution of predicted disorder in Bacteria, Archaea and Eukaryota reflects those obtained in prior studies [8], [9], [10]. Here we collected 194 eukaryotes, 69 bacteria and 18 archaea (for a complete list, cf. Supplementary Table S1, S2 and S3) and predicted structural disorder in all proteins in all proteomes by the IUPred algorithm [21], [22]. From the disorder score, two primary measures of disorder were calculated: the average disorder score values for all proteins averaged for all the proteomes through the entire superkingdom (Figure 1A) and the ratios of disordered residues within the proteins (with a score ≥0.5), also averaged for the proteomes of the entire superkingdom (Figure 1B). (Other measures of structural disorder, such as the ratio of proteins with at least one long (≥30 consecutive disordered residues) disordered region and the ratio of amino acids within these regions, were also calculated (cf. Supplementary Table S1, S2 and S3), but are not plotted because they showed very similar qualitative trends).

Overall, predicted structural disorder shows the trends established earlier, eukaryotes having higher averages than both prokaryotic groups, Bacteria being clearly higher than Archaea [8], [9], [10]. Due to the large number of proteomes, it is now clear that there is no straightforward correlation between disorder and phylogeny, because both prokaryotes and eukaryotes show large variations with extensive overlaps, with all the reference prokaryotes being higher than the lowest of eukaryotes, for example. Therefore, we asked further what possibly distinguishes prokaryotes from eukaryotes, thus we also predicted and compared their number of disordered binding regions by the ANCHOR method [16] (Figure 1C) and the number of different Pfam domain types occurring in their proteomes (Figure 1D). As a first conclusion, all three features seem to distinguish between prokaryotes and eukaryotes to some extent, and their combination might be a strong descriptor of protein phylogeny (a similar conclusion was also reached in [17]).

### Structural disorder in all eukaryotes

**Figure 2:**
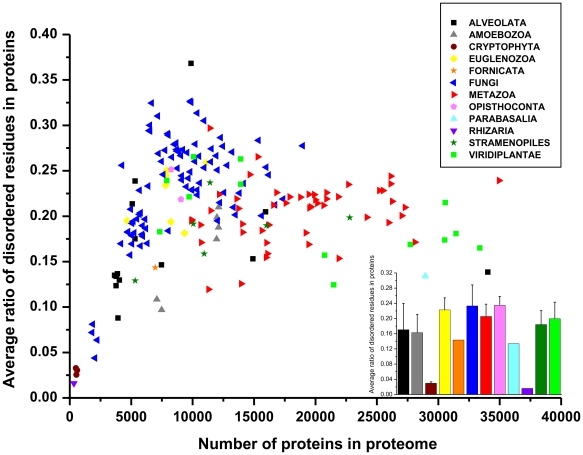
Ratio of disordered residues in the proteins of eukaryotic proteomes. On the main plot the average ratio of disordered residues (with an IUPred score ≥0.5) in proteins of all eukaryotic proteomes is shown as a function of the number of proteins in the given proteome. Large phylogenetic groups are indicated with different colors, as defined on a small plate. In the insert, the average and standard deviation for the different groups is given, by applying the same color code as in the main plot.

Next, we asked about the reason of this lack of clear separation between the large phylogenetic groups. Previous studies of bacterial proteomes have shown large inter-species variations possibly linked with lifestyle and habitat [11], [12], [13]. Here we calculated the ratio of disordered residues for all the 194 eukaryotic proteomes (and also other measures, cf. Supplementary Table S1, showing the same trends). The values plotted as a function of the number of proteins in the proteome (Figure 2) are scattered over a broad range (0.016 to 0.368), and show a general but not strictly monotonous increase with proteome size. Mostly single-celled organisms are responsible for the deviation from linearity, because they cover the entire range, reaching both above and below multicellular organisms (plants and animals). Multicellular organisms consistently have intermediate values, most of them falling between 0.15 and 0.25. In addition, the level of structural disorder in protists is not random but distinct clades show characteristic differences, very much in line with their pathogenic status and lifestyle. Free living and host-changing organisms have the highest levels, whereas endosymbionts and obligate intracellular pathogens completing their life cycle in the same host have the lowest levels of structural disorder (cf. also Discussion).

### Number of Pfam domain families in proteomes

Structural disorder shows very large inter-species variations among eukaryotes, which might also be reflected in – or be a reflection of – the number of types of protein domains in the different species. This expectation is based on IDPs/IDRs often carrying out their function in association with folded domains, either as linkers in multidomain proteins [29], [30] or as motifs/disordered domains [23], [24] binding in and induced folding process to cognate domains [5]. To this end, we asked how the number of distinct Pfam domains (domain types) changes with the number of proteins in proteomes and if it reflects the evolutionary division between prokaryotes and eukaryotes. We predicted the number of distinct Pfam-A families, domains and repeats for every protein in our datasets with the PfamScan search algorithm [28]; summary data for the entire superkingdoms are already shown in Figure 1D.

Unlike structural disorder, the number of domain types shows a monotonous increase with proteome size throughout the superkingdom of eukaryotes, from as low as 200 domains to as high as 4500. The similarities and differences carry important evolutionary and functional information, as addressed below.

### The correlation of structural disorder, domains and short binding motifs

**Figure 3:**
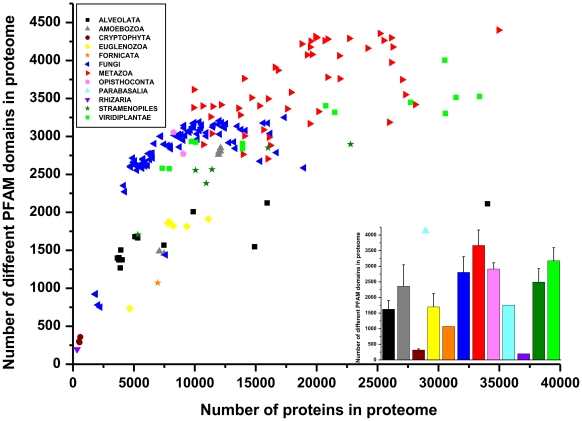
Number of different Pfam domains in eukaryotic proteomes. Pfam domains were predicted for all eukaryotic proteomes with PfamScan [28]. On the main plot the number of different types of Pfam domains is shown as a function of the number of proteins in the given proteome. Large phylogenetic groups are indicated with different colors, as defined on a small plate. In the insert, the average and standard deviation for the different groups are given, by applying the same color code as in the main plot.

**Figure 4:**
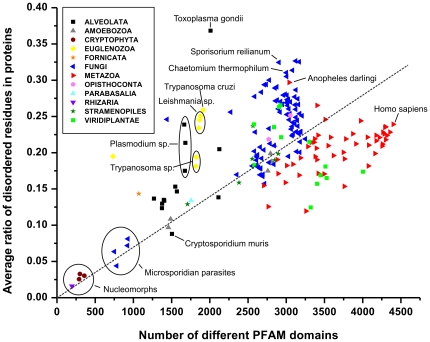
The interplay of structural disorder and Pfam domain types. The average ratio of disordered residues (with a score ≥0.5) in proteins of the eukaryotic proteomes, is shown as a function of the number of different Pfam domains found. Large phylogenetic groups are color coded, as defined on a small plate. The linear fit of the data is shown as a dashed line. Certain species are named and certain groups are encircled and marked, as explained and discussed in the text.

As seen, both structural disorder and the number of Pfam domains in the proteome increase in evolution, roughly in proportion to the number of proteins in the proteomes (Figure 2 and Figure 3, respectively), with apparent and significant differences, though. As suggested in the Introduction, disordered proteins/regions function either as entropic chains (linkers between domains) or via molecular recognition, in which they use either short binding motifs [23] or disordered domains [24]. In this sense, the evolution of domains and disordered regions is intertwined and is often inseparable. To visualize this interdependence, and possible critical differences, we plotted the average ratio of disordered residues as a function of the number of different Pfam domains (Figure 4). The straight line fitted proves a good correlation between the two, however, certain groups show systematic deviations (see also Discussion). Fungi consistently tend to have more disorder than the average, whereas Metazoa have less (interestingly, among all the metazoans, the mosquito, *Anopheles darlingi* has the highest ratio of disordered residues in its proteins. This species of mosquito is the most important malaria vector in South America and it is capable of transmitting both *Plasmodium falciparum* and *Plasmodium vivax*. Whether its high disorder is related to this fact, remains to be seen).

Pathogenic protists, such as *Trypanosoma* and *Leishmania* species (Euglenozoa) or *Plasmodium* species (Alveolata) have much more disorder than expected. Viridiplantae are clearly separated into two groups by the line: multicellular land plants tend to have less disorder, whereas single-celled algae have more disorder than expected. Obligate intracellular parasites, such as Microsporidia (Fungi) and the endosymbiotic nucleomorphs (Cryptophyta and Rhizaria), have very little of both in proportion: reduction of their genomes seems to have shaved all superfluous domains and disordered regions down to the acceptable minimum. Their detailed studies might help understand the types of proteins and functions that cannot exist without structural disorder.

**Figure 5:**
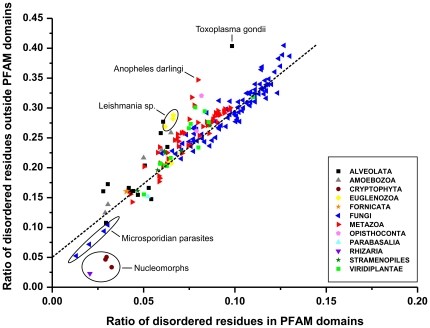
The ratio of structural disorder within and outside Pfam domains. The average ratio of disordered residues (with a score ≥0.5) in proteins of the eukaryotic proteomes, within regions outside Pfam domains is shown as a function the same value within Pfam domains. Large phylogenetic groups are color coded, as defined on a small plate. The linear function showing a parallel increase of disorder within and outside Pfam domains in most species is marked by a dashed line. Certain groups of species show significant deviation from this linear dependence, as explained and discussed in the text.

The parallel increase of the number of domain types and the ratio of disordered residues may be conceived either as a result of an increase of disorder both within and outside domains and also as an increase only in the regions outside domains. Whereas disorder occurs with a notable frequency also within domains [24], we found that regions outside domains are about three times more disordered. To address the correlation of domains and disorder, we have directly calculated the level of disorder within and outside domains (Supplementary Figure S2). The two values show a very strong correlation (Figure 5), i.e. large evolutionary variations of disorder in eukaryotes results from parallel changes in disorder within and outside Pfam domains. There seem to be very little compensatory effects (e.g. low level of disorder in domains compensated by very high levels of disorder outside domains). There are a few significant exceptions, though. Pathogenic protists and *Anopheles darlingi* show significantly higher disorder outside their domains than expected, whereas obligate intracellular parasites Microsporodia and Nucleomorphs have much less. Apparently, these latter organisms have given up on all regulatory functions linked with disorder outside their domains. This feature is also apparent in the almost complete lack of disordered binding regions in them (see next section).

**Figure 6:**
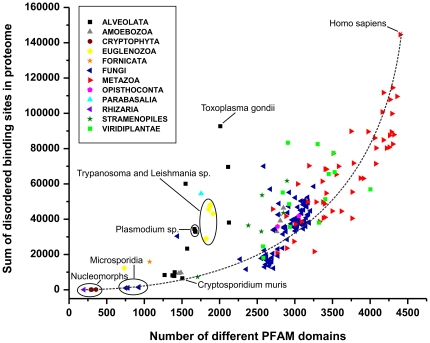
Number of disordered binding sites in eukaryotic proteomes. The overall number of predicted disordered binding sites in eukaryotic proteomes predicted by the ANCHOR algorithm [16] is shown as function of the number of different Pfam domains. Large phylogenetic groups are color coded, as defined on a small plate. Certain species and certain groups are marked and/or named, as explained and discussed in the text. The increasing (exponential) function marked by the dashed line is no fit by any model, it is only drawn to guide the eye. Homo sapiens has a larger apparent proteome size, because it has been analyzed at greater depth and the number of identified isoforms exceeds that of other mammals even after sequence identity filtering.

Turning to recognition motifs directly, it should be noted that these are short (typically 3–15 residues in length [31]), they contain very little sequence information and their prediction from sequence is fraught with extremely high false positive rates. An unbiased prediction of disordered binding sites relies on assessing their interaction energy with a potential partner. We used this algorithm, ANCHOR [16], to predict the number of disordered binding sites in all proteins in all the proteomes (Supplementary Table S1, S2 and S3, already plotted for the superkingdoms, cf. Figure 1C) and plot it as a function of the number of domains (Figure 6). Again, there is an overall correlation between these two measures, which shows and exponential character, with more domains being associated with disproportionately more binding sites. This also somewhat explains the deviations from the linear regression on Figure 4. Fungi and pathogenic *Trypanosoma*, *Leishmania* (Euglenozoa) and *Plasmodium* (Alveolata), which all have more disorder then expected based on the number of their Pfam domains (Figure 4), are clearly distinguished here. Pathogenic organisms use a lot of this “excess” disorder for short binding motifs (cf. also Figure 5), probably involved in pathogen-host interactions, as already suggested for viruses [32]. Fungi, on the other hand, may have more of their disordered regions probably serving as linkers (entropic chains) in large multidomain proteins.

## Discussion

In this paper the first full analysis of structural disorder in all eukaryotic proteomes sequenced thus far is presented. Several general conclusions can be drawn, some corroborate and/or extend previous notions derived from much more limited data, others provide completely new insight into the evolution, distribution and likely functional importance of structural disorder. Our starting point is that this study reaffirms previous assertions that the number of proteins in proteome is not a good measure of complexity [17], confirmed here by the whole body of data (cf. for example the comparable size of the proteome of humans and the ciliated protist *Paramecium tetraurelia*, or plants of very similar complexity that have undergone genome duplications).

Therefore, we concentrated our study on structural disorder, the number of different Pfam domain types and disordered binding motifs in all the eukaryotic proteomes available today. The classical notion of the field is that structural disorder has contributed to the evolutionary transition from prokaryotes to eukaryotes [8], [9], [10]. By comparing 194 eukaryotic genomes to 69 (bacterial)+18 (archaeal) reference prokaryote genomes, we do find that this view is largely correct, but only with serious reservations. Prokaryotes do have a lower average disorder than eukaryotes (e.g. mean disorder score, bacteria: 0.211, archaea: 0.185, eukaryotes: 0.30), but both large groups extend over a very broad range of predicted disorder values (prokaryotes: 0.12 to 0.35, eukaryotes: 0.1 to 0.41). The view that eukaryotes contain more disorder than prokaryotes is an oversimplification, meaning that structural disorder does not simply depend on the complexity of the organism and does not define the phylogenetic group to which the organism belongs [17]. Rather, it represents a rapidly evolving modality which contributes to the fast appearance of novel functions, as already suggested for all prokaryotes [11] and archaea [13]. A decreased level of disorder seems to reflect adaptation to extreme conditions [11], or a nutrient-rich, stress-free (lenient [33]) intracellular environment, whereas an elevated level indicates that the organism leads a varied lifestyle in which it can change between habitats, most apparent in the case of host-changing pathogens. A comparison of superkingdoms suggests that the number of disordered binding sites per protein and the number of different Pfam domains is also significantly different between prokaryotes and eukaryotes. Their range of values (average number of disordered binding sites, prokaryotes: 0.072 to 2.93, eukaryotes: 0.11 to 9.4; Pfam domain types, prokaryotes: 376 to 2417, eukaryotes: 196 to 4400) separate large superkingdoms better, showing significantly less overlap.

The comparison of the different measures provide further insight: the number of domain types shows a rather monotonous increase in evolution, whereas the level of disorder shows an overall increase but large evolutionary fluctuations in certain clades, first of all in protists. This difference probably reflects that the creation of novel domain types is very demanding and is an event of low probability: multiple mutations within a sufficiently long region have to accumulate for a stable and functional fold to arise [34]. In accord, the complement of domain types available within a phylogenetic group does not fluctuate on a short timescale. Disorder seems to follow an entirely different path. IDPs evolve fast, accepting many more point mutations, insertions and deletions than globular proteins [35]; even repeat expansions are often observed in IDPs [36]. Its length-distribution follows a power-law (in humans, but probably also in other species), with many short regions but also a significant incidence of very long disordered regions [37]. In contrast with domains, which have been created steadily during evolution [38], structural disorder has the capacity of rapid appearance and disappearance, much contributing to evolutionary innovation, even on relatively short time-scales [39]. Intriguingly, the strict proportionality of disorder within and outside domains shows that disorder does have the potential to invade domains, probably increasing their functional diversity even within one family.

Further complicating their seeming independence is that structural disorder and domains cannot exist and function without each other. For example, multidomain proteins cannot exist without disordered linkers, as seen in the case of scaffold proteins [29]. Structural disorder is also often involved in molecular recognition, when a short disordered motif [31], [32] or even domain [24] undergoes induced folding in the presence of a partner, which is almost invariably a folded domain [5]. Due to this, the two basic types of function of disordered proteins (entropic chains and binding motifs) seem to diverge in this sense, because the number of motifs (that require domains as binding partners) follows pretty closely the expansion of domain families, probably because their reasonable number and varieties is limited by the number of cognate domains they can bind to. This is not the case of motifs that pathogens use to interact with their host [32] and also not for IDPs that function as entropic chains, which have functions either independent of domains [40] or their length is not limited by the domains connected [29], [30], [37].

Therefore, the simple evolutionary trends result in distinct taxon-specific combinations of the number and actual types of domains and the ratio of disorder, as apparent from many observations in our study. Large and complex organisms, such as metazoans and plants have a high and rather even level of disorder. Fungi are more disordered than Metazoa or the Viridiplantae, and they also show more variation. Microsporidian intracellular parasites showing extreme small genome size and very low amount of structural disorder and certain plant pathogens which have very high amount of structural disorder, like the maize pathogen *Sporisorium reilianum*. Among single-celled eukaryotes (protists), on the other hand, we also find the less disordered organisms (Cryptophyta, which are endosymbionts) and the most disordered ones (one Alveolata – Toxoplasma gondii –, and some fungi). It is apparent that obligate parasites and symbionts (the most extreme being endosymbiotic plastids, nucleomorphs) have delegated many of their genes/functions to the host, and have undergone a tremendous genome reduction, this we have also seen in the thermal adaptation of bacteria [11]. On the other hand, free-living organisms, which have to change habitat and have to respond to varying environmental challenges, always have a very high level of disorder. This is also the case with host-changing parasites, rapid evolutionary adaptation of which occurs by creating structural disorder, as already witnessed in the case of apicomplexan parasites [14] and some early-branching pathogenic and non-pathogenic protists [15]. This probably explains why the species show a very high amount of disorder in the *Trypanosoma* genus, these have a complex life cycle and host-changing pathogenic life style. The Alveolata *Cryptosporidium muris*, which does not require a vector and is capable of completing its life cycle within a single host, has low ratio of disorder in its proteome (cf. Figure 4). On the other hand, the absolute recorder is the apicomplexan parasite *Toxoplasma gondii*, because more than 65% of its proteins have at least one long (≥30 consecutive residues) disordered region (Supplementary Table S1). Probably the complex life cycle and the broad range of mammalian hosts represent such a variable environment for this parasite that demands its high amount of disorder and fast rate of evolution. Among the unicellular organisms the Alveolata kingdom shows the highest deviation in disorder content. Many of them have a very high average protein size as well (Supplementary Figure S1), so it seems that their proteins abound in long disordered regions.

The case of pathogens can be rationalized (as also suggested for viruses [32], [41], [42]) by four, somewhat opposing, challenges these organisms face: i) they have to evade the immune system of the host, ii) they have to effectively interact with the host for invasion and iii) also for deregulating host metabolism for their own purposes, and iv) they have to do it with as compact a genome as possible. In these, the advantages of structural disorder are clearly apparent in the pathogenic protists [15]. In addition, the large variation of structural disorder in protists can also be viewed as an evolutionary relic of the role structural disorder played in the rise of eukaryotic organisms, because several novel disorder-related protein functional groups appeared early in eukaryotes, such as transcription factors [43], signaling proteins [29], transmembrane receptors [44], cytoskeletal proteins [45], [46], [47], proteins involved in membrane trafficking [48] and chromatin organization [49], [50], [51]. Further, hub proteins involved in multiple protein-protein interactions, thought to be critical in organizing interactomes of complex organisms, also have been noted for their elevated disorder [52], [53], [54].

In conclusion, we have selected and analyzed structural disorder in 194 genomes, far more than it was possible in previous comparative studies [8], [9], [10]. Our studies reaffirm that structural disorder distinguishes eukaryotes from prokaryotes, but its frequency in both large groups spans a very wide and overlapping range, and prokaryotes can only be clearly separated from eukaryotes if the number of disordered binding regions and different Pfam domain types are also taken into consideration. Extremes of predicted disorder are found in protists, where it correlates strongly with lifestyle. Low levels are observed in obligate intracellular parasites and symbionts, whereas high levels are observed in host-changing parasites. Our interpretation of these and many other particular observations is that protists have been – and still are – the evolutionary hot-bed of experimentation with structural disorder, resulting in rapid adaptive changes in response to environmental challenges.

## Supporting Information

Figure S1 **Structural disorder and protein length in Eukaryotes.** The average ratio of disordered residues (with a score ≥0.5) in proteins of the eukaryotic proteomes, is shown as a function of the average length of proteins in the given proteome. Large phylogenetic groups are color coded, as defined on a small plate. The oval indicates that most species fall within a central range. Certain pathogenic and endosymbiotic species named fall outside, either because they have very long proteins or lower than average disorder.
(TIF)

Figure S2 **Structural disorder within and outside Pfam domains.** The average ratio of disordered residues (with a score ≥0.5) in proteins of the eukaryotic proteomes is calculated separately for regions identified as Pfam domains (**A**) and regions outside Pfam domains (**B**). Large phylogenetic groups are color coded, as defined on a small plate. The linear function showing a parallel increase of disorder within and outside Pfam domains in most species is shown as a dashed line.
(TIF)

Table S1 **Calculated data for Eukaryota.** All the calculated data mentioned in methods for every eukaryotic taxon is included.
(XLS)

Table S2 **Calculated data for Bacteria.** All the calculated data mentioned in methods for every bacterial taxon is included.
(XLS)

Table S3 **Calculated data for Archaea.** All the calculated data mentioned in methods for every archaeal taxon is included.
(XLS)
