# Supplementary figures and images for: Structural Disorder in Eukaryotes
Source: PLoS One. 2012 Apr 5;7(4):e34687. doi: 10.1371/journal.pone.0034687 (PMC3320622; doi:10.1371/journal.pone.0034687)

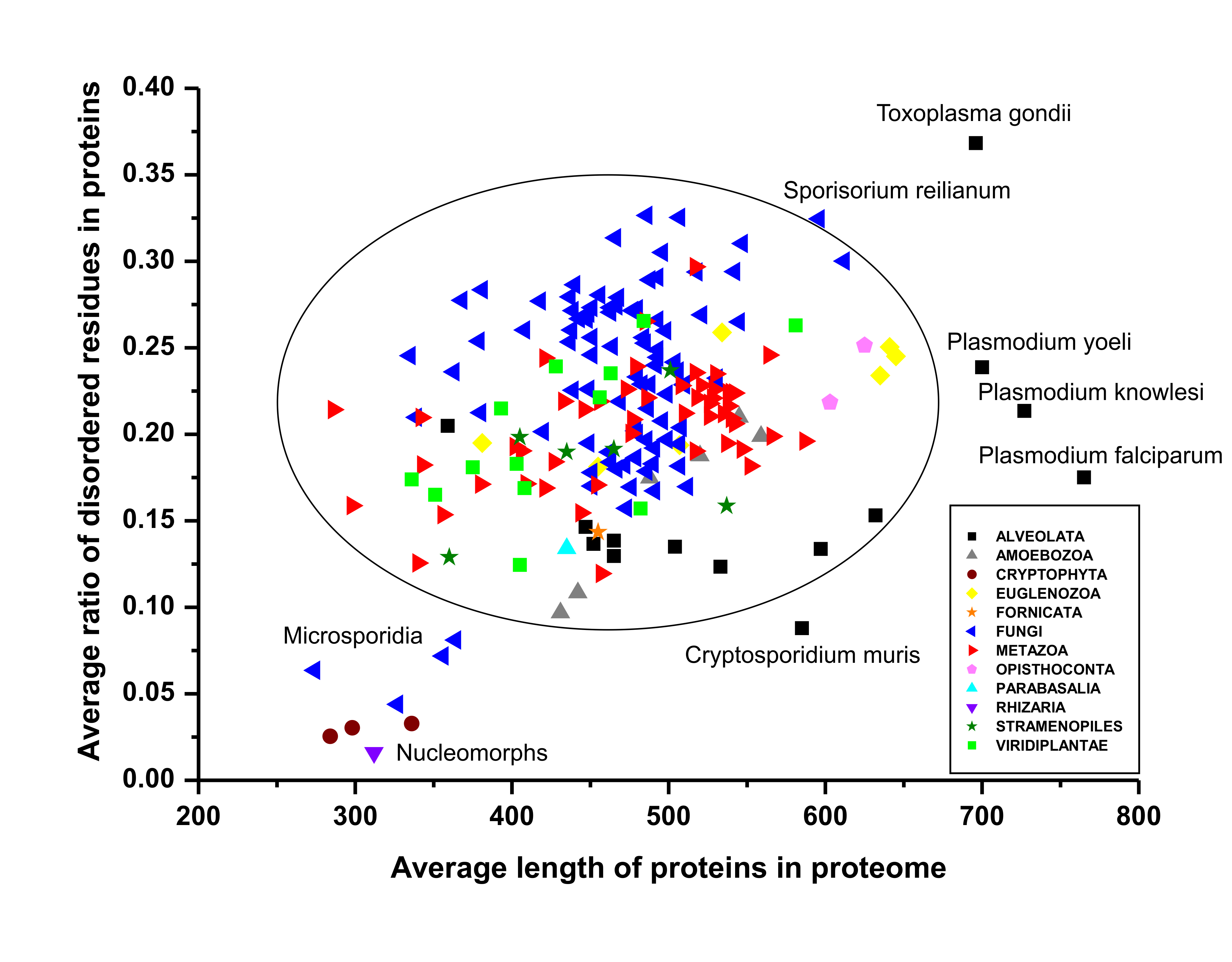

Supplement: Figure S1 — Structural disorder and protein length in Eukaryotes. The average ratio of disordered residues (with a score ≥0.5) in proteins of the eukaryotic proteomes, is shown as a function of the average length of proteins in the given proteome. Large phylogenetic groups are color coded, as defined on a small plate. The oval indicates that most species fall within a central range. Certain pathogenic and endosymbiotic species named fall outside, either because they have very long proteins or lower than average disorder. (TIF) [file pone.0034687.s001.tif]

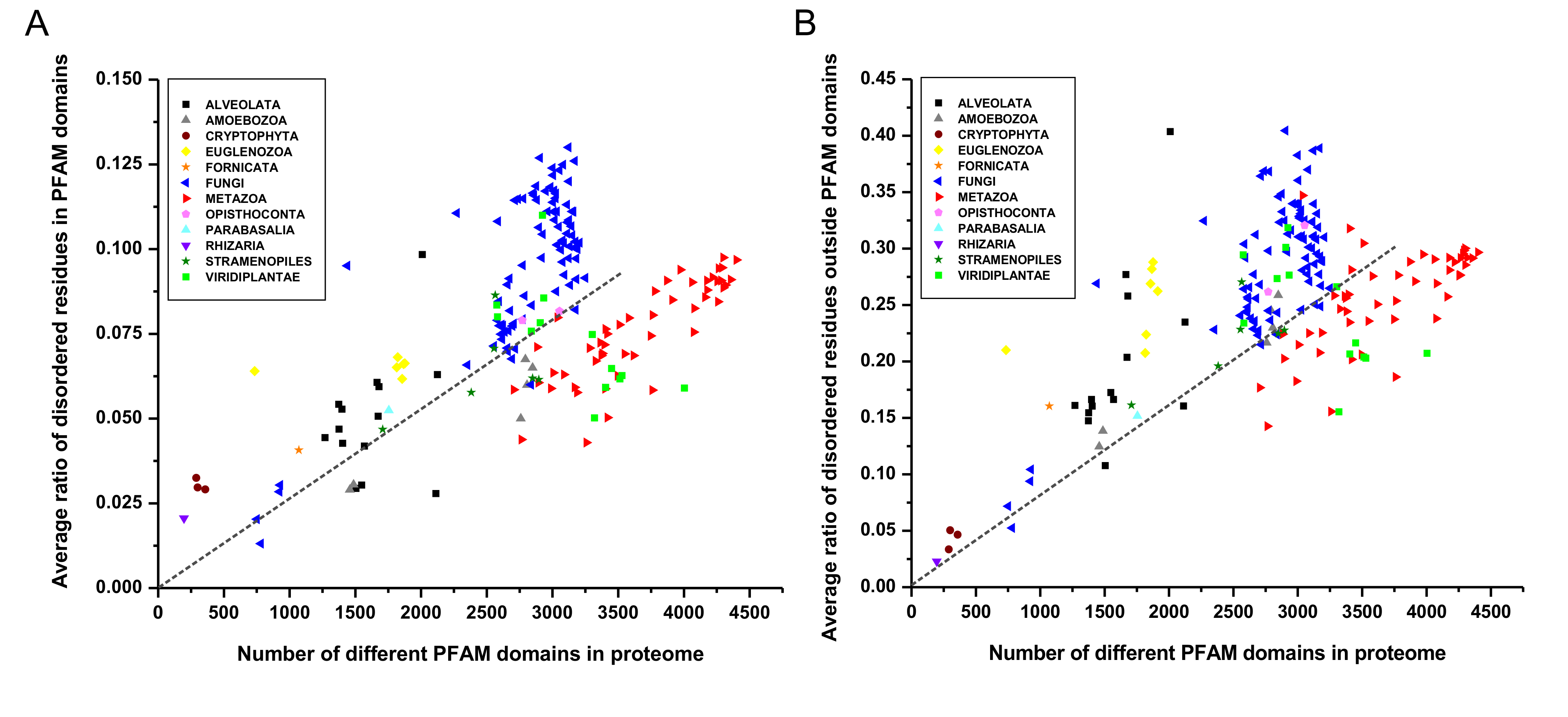

Supplement: Figure S2 — Structural disorder within and outside Pfam domains. The average ratio of disordered residues (with a score ≥0.5) in proteins of the eukaryotic proteomes is calculated separately for regions identified as Pfam domains (A) and regions outside Pfam domains (B). Large phylogenetic groups are color coded, as defined on a small plate. The linear function showing a parallel increase of disorder within and outside Pfam domains in most species is shown as a dashed line. (TIF) [file pone.0034687.s002.tif]
